# Visuospatial memory impairment as a potential neurocognitive marker to predict tau pathology in Alzheimer’s continuum

**DOI:** 10.1186/s13195-021-00909-1

**Published:** 2021-10-09

**Authors:** Eun Hyun Seo, Ho Jae Lim, Hyung-Jun Yoon, Kyu Yeong Choi, Jang Jae Lee, Jun Young Park, Seong Hye Choi, Hoowon Kim, Byeong C. Kim, Kun Ho Lee

**Affiliations:** 1 Gwangju Alzheimer’s Disease and Related Dementia Cohort Research Center, Chosun University, 61452 Gwangju, Republic of Korea; 2 Premedical Science, College of Medicine, Chosun University, Gwangju, 61452 Republic of Korea; 3 Department of Integrative Biological Science, Chosun University, Gwangju, 61452 Republic of Korea; 4 Department of Neuropsychiatry, College of Medicine, Chosun University, Gwangju, 61452 Republic of Korea; 5 Department of Public Health Sciences, Graduate School of Public Health, Seoul National University, Seoul, 08826 Republic of Korea; 6 Department of Neurology, Inha University School of Medicine, Incheon, 22212 Republic of Korea; 7 Department of Neurology, Chosun University Hospital, Gwangju, 61452 Republic of Korea; 8 Department of Neurology, Chonnam National University Medical School, Gwangju, 61469 Republic of Korea; 9 Department of Biomedical Science, Chosun University, Gwangju, 61452 Republic of Korea; 10 Aging Neuroscience Research Group, Korea Brain Research Institute, Daegu, 41062 Republic of Korea; 11 Neurozen Inc., Seoul, 06236 Republic of Korea

**Keywords:** Alzheimer’s disease, ATN classification, Cerebrospinal fluid, Tau, Visuospatial memory, Biomarkers

## Abstract

**Background:**

Given that tau accumulation, not amyloid-β (Aβ) burden, is more closely connected with cognitive impairment in Alzheimer’s disease (AD), a detailed understanding of the tau-related characteristics of cognitive function is critical in both clinical and research settings. We investigated the association between phosphorylated tau (p-Tau) level and cognitive impairment across the AD continuum and the mediating role of medial temporal lobe (MTL) atrophy. We also developed a prediction model for abnormal tau accumulation.

**Methods:**

We included participants from the Gwangju Alzheimer’s Disease and Related Dementia Cohort in Korea, who completed cerebrospinal fluid analysis and clinical evaluation, and corresponded to one of three groups according to the biomarkers of A and T profiles based on the National Institute on Aging and Alzheimer’s Association research framework. Multiple linear and logistic regression analyses were performed to examine the association between p-Tau and cognition and to develop prediction models. Receiver operating characteristic curve analysis was performed to examine the discrimination ability of the models.

**Results:**

Among 185 participants, 93 were classified as A-T-, 23 as A+T-, and 69 as A+T+. There was an association between decreased visuospatial delayed memory performance and p-Tau level (*B* = − 0.754, *β* = − 0.363, *p* < 0.001), independent of other relevant variables (e.g., Aβ). MTL neurodegeneration was found to mediate the association between the two. Prediction models with visuospatial delayed memory alone (area under the curve [AUC] = 0.872) and visuospatial delayed memory and entorhinal thickness (AUC = 0.921) for abnormal tau accumulation were suggested and they were validated in an independent sample (AUC = 0.879 and 0.891, respectively).

**Conclusion:**

It is crucial to identify sensitive cognitive measures that capture subtle cognitive impairment associated with underlying pathological changes. Preliminary findings from the current study might suggest that abnormal tau accumulation underlies episodic memory impairment, particularly visuospatial modality, in the AD continuum. Suggested models are potentially useful in predicting tau pathology, and might be utilized practically in the field.

**Supplementary Information:**

The online version contains supplementary material available at 10.1186/s13195-021-00909-1.

## Background

Biomarker identification for Alzheimer’s disease (AD) has evolved over the past few decades. Accordingly, the research framework for AD has changed dramatically [1]. It is moving from a clinically defined to a biologically defined disease that is understood to begin considerably earlier than the onset of cognitive decline. This framework enables us to define the earliest stages of the disease continuum. In addition, with a biomarker-based definition of AD, the mechanisms underlying the clinical manifestations of AD can be understood more precisely. Clinically observed cognitive symptoms are not always specific to AD. However, the characteristics of cognitive impairment that are closely associated with AD neuropathological processes can be explored.

Amyloid-beta (Aβ) plaques and tau neurofibrillary tangles (NFT) are known to be the hallmarks of AD pathology, and they begin to accumulate in the brain years before clinical symptoms [2]. Aβ deposition, pathologic tau, and neurodegeneration have been served as biomarkers to define AD, which lead AT(N) classification system [1]. Moreover, many studies using cerebrospinal fluid (CSF) demonstrated such classification or combination of the three biomarkers have prognostic utility as well as diagnostic value [3–5]. In addition, ratio between Aβ_1–42_ and phosphorylated tau (p-Tau) also showed association with subsequent cognitive decline in cognitively normal (CN) or mild cognitive impairment (MCI) older adults [3, 5]. Over the past decade, accumulating evidence has suggested that early cognitive changes are more closely associated with tau pathology than Aβ pathology. Transgenic mouse model [6], clinicopathological [7], and neuroimaging [8–10] studies support that Aβ is the initial factor, and clinical symptoms begin to appear as tau pathology progresses. Especially, a number of studies have reported that tau accumulation could be a highly predictive indicator for future cognitive decline in both nondemented [8, 11] and AD [12]. Given that tau accumulation, not Aβ burden, is more closely connected with cognitive change in the AD continuum [13–15], a detailed understanding of the relationship between tau accumulation and specific cognitive function is critical for effectively capturing AD-related symptoms at the earliest stage of the disease.

Several recent studies have attempted to determine whether there are tau-related cognitive impairments, but their findings have been inconclusive. Many studies have reported associations between tau pathology and episodic memory [8, 11, 16, 17]. For example, two studies using CSF p-Tau demonstrated significant associations between episodic memory and tau levels [17, 18]. Studies using tau positron emission tomography (PET) also reported that elevated tau deposition in the medial temporal lobe (MTL) was associated with episodic memory impairment [8, 9]. However, other studies have shown a significant association between tau pathology and executive function, not episodic memory [19], or no association with any of the cognitive domain [20, 21]. The specific tau-associated cognitive symptoms in individuals with AD continuum remain unclear.

Although several attempts have been made to shed light on tau-associated cognitive impairment in aging populations, previous studies have had several limitations. Some studies have used relatively brief cognitive tests [18, 21] or only memory indexes [22]. Visuospatial memory tests were not included in most previous studies [8–10, 16, 18–22]. More comprehensive neuropsychological measures that assess multiple cognitive domains should be utilized. In addition, in some studies, tau-associated cognitive impairment was investigated without controlling for the influence of Aβ pathology [9, 16]. Due to concomitant tau and Aβ pathology, the influence of Aβ pathology on cognitive function should be controlled to investigate tau-specific cognitive impairment.

Given that NFT initially forms in MTL regions [23, 24] and that tau pathology is closely linked to longitudinal cortical thinning, especially in MTL [25], atrophy in such regions could play a role in cognitive impairment. One recent investigation reported both direct and indirect (i.e., gray matter-mediated) tau effects on cognition [26]. Although tau-associated cognitive impairment could be mediated by gray matter loss in such brain structures, only a few studies have investigated this mediated association.

Tau accumulation is generally measured by either tau PET or CSF p-Tau levels. However, these measurements are not always feasible, especially in community-based settings. Noninvasive and cost-effective markers with good predictability for abnormal tau accumulation, i.e., biomarker “T” positive, are highly desirable. However, to date, few studies have explored prediction models for abnormal tau accumulation based on clinical information that is relatively easy to obtain.

Therefore, we aimed to investigate the specific association between p-Tau level and cognitive function across the AD continuum and to examine the mediating effects of MTL neurodegeneration on this association. We also developed prediction models for abnormal tau accumulation to facilitate the use of specific neuropsychological tests in the field. Finally, the validities of the prediction models were tested on an independent dataset.

## Methods

### Participants

This is a cross-sectional study. We recruited individuals who had agreed to undergo lumbar puncture in a pool of older adults registered at the Gwangju Alzheimer’s Disease and Related Dementia (GARD) Cohort from December 2014 to October 2019. The GARD database has been described previously [27, 28]. Briefly, inclusion criteria in GARD were as follows: for CN participants (1) aged 60 and more, (2) a clinical dementia rating (CDR) score of 0, (3) a normal range of cognitive function, i.e., all neuropsychological tests z-scores were above − 1.5 standard deviation (SD) according to age-, education-, and gender-specific norms; for MCI (1) aged 60 and more, (2) a CDR score of 0.5, (3) meet MCI criteria by Winblad [29]; and for AD dementia (1) a CDR score of 0.5 and more, (2) meet the Diagnostic and Statistical Manual of Mental Disorders (DSM-IV) criteria for dementia [30] and the National Institute of Neurological and Communication Disorders and Stroke-Alzheimer’s Disease and Related Disorders Association criteria for probable AD [31]. Exclusion criteria in GARD were applied as follows: (1) illiteracy; (2) severe vision or hearing loss; (3) evidence of focal brain lesions on MRI other than suspected incipient AD; (4) any significant neurologic, medical, or neuropsychiatric disorders (e.g., depression or anxiety disorders) that could affect mental function; and (5) current use of psychoactive medications. The institutional review board of Chosun University Hospital and Chonnam National University approved the study. Written informed consent was obtained from each participant or his or her legal guardian. This study was conducted in accordance with the Declaration of Helsinki.

### CSF collection and analysis

CSF was obtained by lumbar puncture with aseptic technique at the L3–L4 or L4–L5 intervertebral spinous process space, using a 22- or 21-gauge needle, and collected in Falcon polypropylene tubes (BD Biosciences, Franklin Lakes, NJ, USA). All CSF samples were analyzed using the Luminex 200 MAP system (INNO-BIA AlzBio3 for research-only reagents, Fujirebio, Ghent, Belgium). Samples were taken from the deep freezer 4 h before use and continuously kept on ice. After pre-washing the 96-well filter plate for vacuum with a 1/25 diluted wash buffer, 25 μL of each microsphere binding the corresponding ATN biomarker-specific capture antibodies (4D7A3, AT270, and AT120 for Aβ_1–42_, p-Tau_181_, and total tau protein [t-Tau], respectively) were bound with biotinylated monoclonal antibodies and capture antibodies (3D6 for Aβ_1–42_, and HT7 for p-Tau_181_ and t-Tau). Standard curves were constructed for ATN biomarkers using a sigmoidal curve-fitting, and the fluorescence intensity of the mean values for the duplicate samples were obtained with the concentration of ATN biomarkers. The cut-off values for the ATN biomarkers were determined using the Youden index method. Positive values were defined as follows (pg/mL): an “A” biomarker of Aβ_1–42_ < 385.822, “T” biomarker of p-Tau_181_ > 41.881, and “N” biomarker of t-Tau > 78.996. Abnormal tau accumulation was designated based on “T” biomarker of p-Tau_181_ > 41.881.

### Classification of A and T biomarker profiles

There were 359 individuals who completed CSF analysis in the GARD database. Of these, we excluded candidates with evidence of focal brain lesions on MRI other than suspected incipient AD (*n* = 8); any type of dementia other than AD (*n* = 8); any significant neurologic, medical, or psychiatric disorders that could affect mental function (*n* = 19); no amyloid PET (*n* = 17); or inconsistent Aβ positivity between PET and CSF analysis (*n* = 48); or no neuropsychological test scores available (*n* = 4). In addition, individuals were excluded if the interval between lumbar puncture and neuropsychological assessment was > 6 months (*n* = 38). Among the remaining 217 individuals 57 were CN older adults, 111 had MCI, and 49 had AD.

**Fig. 1:**
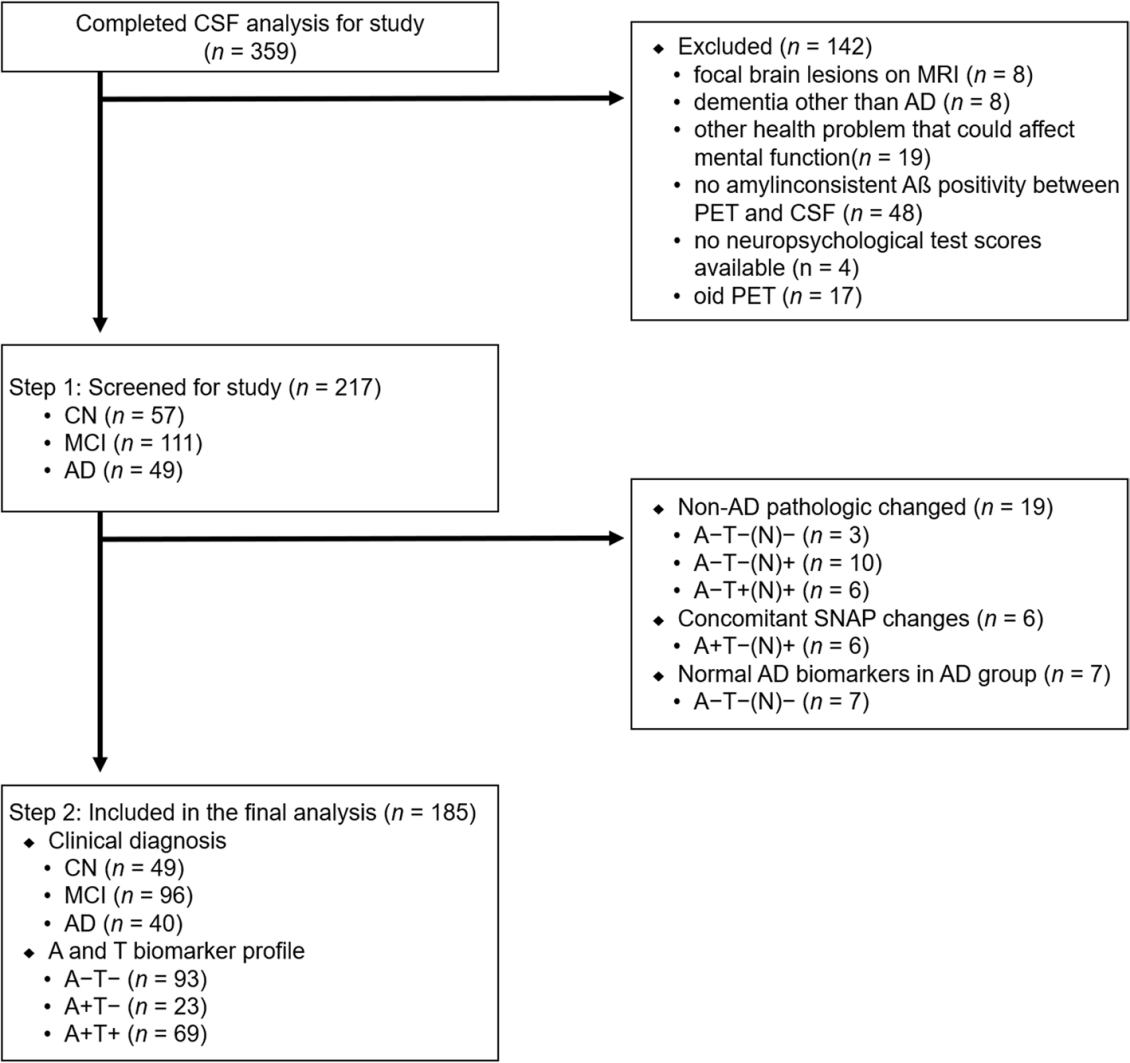
Flowchart of study sample recruitment. ATN classification system (amyloid, tau, neurodegeneration), for “A” (CSF Aβ_1–42_), “T” (CSF p-Tau_181_), and “(N)” (CSF t-Tau). CSF, cerebrospinal fluid; CN, cognitive normal; MCI, mild cognitive impairment; AD, Alzheimer’s disease; PET, positron emission tomography; MRI, magnetic resonance imaging; SNAP, suspected non-Alzheimer’s disease pathophysiology

Based on the National Institute on Aging and Alzheimer’s Association research framework [1], we further excluded individuals with non-AD pathologic changes, that is, A-T+(N)- (*n* = 3, 1 CN, 1 MCI, and 1 AD), A-T-(N)+ (*n* = 10, 2 CN and 8 MCI), and A-T+(N)+ (*n* = 6, 3 CN and 3 MCI). Individuals with Alzheimer’s disease and concomitant suspected non-Alzheimer’s pathologic changes, that is, A+T-(N)+ (*n* = 6, 2 CN, 3 MCI, and 1 AD), and individuals with normal AD biomarkers in the AD group, that is, A-T-(N)- (*n* = 7) were also excluded. Therefore, 49 CN individuals, 96 with MCI, and 40 AD dementia were included in the final analysis (Fig. 1) (Supplementary Table S1).

The neurodegeneration biomarker N is not specific for AD and cannot define the AD continuum [1, 32]. Many other conditions, such as aging and cerebrovascular damage, can contribute to neurodegeneration [32–34]. Furthermore, t-Tau shows high correlation with p-tau [35] indicating little additive value of t-Tau when making a diagnosis and prognosis of AD [36, 37]. Therefore, we categorized groups based only on A (based on the cutoff of Aβ_1–42_) and T (based on the cutoff of p-Tau_181_) positivity. The participants were divided into three groups according to their A and T biomarker profiles: A-T-, A+T-, and A+T+.

### Clinical and neuropsychological assessments

Information on participants’ demographic characteristics and medical history were collected. The Geriatric Depression Scale [38] and Korean version of the Mini-Mental State Examination (K-MMSE) [39] were administered. In addition, a comprehensive neuropsychological assessment was performed. The psychomotor speed was assessed using trail making test part A (TMT A). The attention domain was assessed using the digit span forward and digit span backward tasks. The language domain was assessed using a shortened version of the Boston Naming Test (15-item version, form A). The visuospatial domain was assessed with the copying test from the Rey complex figure test (RCFT copy). The memory domain was assessed with six measures: the Seoul Verbal Learning Test (SVLT) immediate recall (SVLT imm), 20-min delayed recall (SVLT delayed), and yes-no recognition (SVLT rec), and the RCFT immediate recall (RCFT imm), 20-min delayed recall (RCFT delayed), and yes-no recognition (RCFT rec). For 61% (*n* = 113) of participants, the Logical Memory (LM) subtest of the 4th edition of the Wechsler Memory Scale [40] was additionally administered. It consists of three parts: immediate recall (LMI), delayed recall (LMII), and delayed recognition (LM rec). Executive function was assessed using the animal fluency test, a phonemic fluency test, the Stroop test (color naming in color-word incongruent conditions), and TMT B.

### Determination of apolipoprotein ε4 genotype

The procedure for determining the apolipoprotein (APOE) genotype has been previously described [41]. Briefly, genomic DNA was extracted from buffy coats isolated from whole blood, and the APOE genotype was determined by single-nucleotide polymorphisms of rs429358 and rs7412. The APOE ε4 positive genotype was assigned if at least one ε4 allele was present.

### Imaging acquisition and processing

MRI was performed using a 3.0 T MR scanner (Skyra, Siemens; 20-channel head coil; MRAGE sagittal view; TR = 2300 ms; TE = 2.143 ms; TI = 900 ms; FA = 9°; FoV = 256 mm × 256 mm, matrix = 320 × 320, and slice thickness = 0.8 mm). MRI data from 11 participants were excluded from the analysis because they were scanned using 1.5 T. The volumes of cortical and subcortical structures were measured from each brain image using the standard recon-all processing pipeline of FreeSurfer version 5.3.0 (http://surfer.nmr.mgh.harvard.edu/). The automated reconstruction protocol has been described previously [42, 43]. We selected two regions of interest (ROIs) from the MTL including the hippocampus (HC) volume and entorhinal cortex (EC) thickness, and intracranial volume (ICV). Additionally, MRI ROIs outside MTL region including lingual gyrus, thalamus, and orbitofrontal area also obtained as control ROIs to confirm discriminative validity. ROIs were obtained using Aseg Atlas (https://surfer.nmr.mgh.harvard.edu/ftp/articles/fischl02-labeling.pdf) [44], and the Desikan-Killiany-Tourville atlas [45].

### Validation of the prediction model

We tested the validity of the prediction model for abnormal tau accumulation, using an independent dataset. The validation dataset was obtained from the validation cohort of the Korean Brain Aging Study for Early Diagnosis and Prediction of AD (KBASE-V) [46]. We included CSF data and compatible clinical information, such as neuropsychological test scores and MRI, where available. The validation dataset consisted of 49 A-T-, 5 A+T-, and 17 A+T+ (Supplementary Table S2).

### Statistical analysis

Demographic characteristics and clinical information were compared among groups using separate one-way analyses of variance (ANOVA) and *χ*^*2*^ tests for continuous and categorical variables, respectively. MTL ROIs were compared among groups, controlling for ICV using analyses of covariance. Analyses of covariance were also performed to compare neuropsychological scores, controlling for age, education, and gender. We controlled demographic variables because neuropsychological test performance in Korean older adults strongly influenced by them [47]. To control for type I errors, a Bonferroni correction was carried out. When the results of the analyses of covariance were significant, a pairwise Bonferroni post hoc test was applied. Partial correlations between the p-Tau level and neuropsychological scores controlling for age, gender, education, APOE ε4 positivity, K-MMSE, and Aβ_1–42_ levels were performed. For neuropsychological scores that showed significant group difference, we performed hierarchical multivariable linear regression analysis. In the first step, age, gender, education, APOE ε4 positivity, K-MMSE, and Aβ_1–42_ levels were entered. These variables were considered as control variables; therefore, they were entered with “enter” method. Then, neuropsychological scores were entered with “stepwise selection” method in order to identify specific neuropsychological measures that associated with p-Tau level. To test whether MTL atrophy (HC, EC) and other control ROIs mediated the association between these two, multiple mediation analysis was performed using 10,000 bootstrapping samples and 95% confidence intervals (CIs) [48]. Age, gender, education, and ICV were entered as covariates in the mediation analysis. Finally, a series of logistic regression analyses were conducted to develop a prediction model for abnormal tau accumulation. The predictors were entered into the models with adjustments for the demographic variables, and ICV was adjusted for MTL ROIs. To compare the predictability among the various models, we used the differences of log likelihood (-2LL). The -2LL is directly proportional to the contribution of variables to the separation of groups, and a smaller -2LL indicates better predictability of the model [49]. After selecting the optimal models, receiver operating characteristic (ROC) curve analysis was performed to examine the discrimination ability of the model. In addition, the prediction model was applied to an independent sample (validation dataset) to validate the model. Additional ROC curve analysis was performed if suggested models also sufficiently discriminate clinical status (CN, MCI, and AD dementia). These analyses were performed using SPSS version 25.0, and the PROCESS macro for SPSS (IBM Corporation, Armonk, NY, USA). A *p*-value less than 0.05 was considered statistically significant.

## Results

### Participant characteristics

Independent of the clinical diagnosis, 185 participants were divided into three groups based on the biomarker combination of A and T positivity. Ninety-three participants were classified as having normal AD biomarkers (A-T-), 23 were found to have Alzheimer’s pathologic changes (A+T-), and 69 had AD (A+T+). There were no group differences in age, education, gender, or subjective depression levels. APOE *ε4* carriers were more frequent in the A+T- and A+T+ groups than in the A-T- group (*p* < 0.001). Aβ_1–42_ levels were significantly lower in the A+T- and A+T+ groups than in the A-T- group (*p* < 0.001). p-Tau and t-Tau were significantly higher in the A+T+ group than in the other two groups (*p* < 0.001). K-MMSE score and MTL ROIs values in the A+T+ group were significantly lower in A+T+ group than in A-T- and A+T-, while there was no significant difference between A-T- and A+T- (Table 1). The mean time between CSF sampling and neuropsychological assessment was 2.75 ± 1.58 months and between CSF sampling and MRI scan was 2.32 ± 2.37 months.

**Table 1:** Demographic and clinical characteristics of study participants

	A-/T- (*n* = 93)	A+/T- (*n* = 23)	A+/T+ (*n* = 69)	***p*** value
**Age**	70.3 ± 6.3	71.3 ± 6.5	69.5 ± 9.0	0.606
**Education**	10.4 ± 5.0	9.8 ± 5.8	9.1 ± 4.6	0.249
**Female,** ***n*** **(%)**	46 (49.5)	13 (56.5)	36 (52.2)	0.820
**APOE ε4 carrier,**^**a**^ ***n*** **(%)**	16 (17.2)	17 (77.3)	42 (60.9)	< 0.001
**MMSE**	26.6 ± 2.6	25.0 ± 4.5	22.4 ± 5.4^*†^	< 0.001
**GDS**	10.6 ± 6.9	9.6 ± 6.6	10.6 ± 6.5	0.822
**CN/MCI/AD,** ***n***	41/52/0	6/12/5	3/31/35	< 0.001
**CDR**^a^ 0, *n* (%)	41 (44.1)	7 (30.4)	3 (4.3)	< 0.001
0.5, *n* (%)	52 (55.9)	15 (65.2)	43 (62.3)	
1, *n* (%)	0 (0.0)	1 (4.3)	22 (31.9)	
**CSF biomarkers** (pg/ml)				
Aβ_1–42_	516.8 ± 98.4	224.5 ± 104.4^*^	235.2 ± 88.9^*^	< 0.001
p-Tau_181_	29.3 ± 5.5	31.2 ± 5.8	60.2 ± 13.2^*†^	< 0.001
t-Tau	49.6 ± 13.1	48.6 ± 16.0	108.7 ± 40.2^*†^	< 0.001
**MTL ROIs**^b^				
L. HC	3951 ± 50	3675 ± 105	3215 ± 61^*†^	< 0.001
R. HC	4202 ± 57	4002 ± 118	3498 ± 69^*†^	< 0.001
L. EC	3.28 ± 0.04	3.28 ± 0.08	2.79 ± 0.05^*†^	< 0.001
R. EC	3.52 ± 0.04	3.52 ± 0.09	3.11 ± 0.05^*†^	< 0.001

Data are presented as means ± standard deviations, unless specified otherwise. A and T classification system, for “A” (based on the value of CSF Aβ_1–42_), and “T” (based on the value of CSF p-Tau_181_). The following CSF thresholds were used: 385.822 pg/mL for Aβ_1–42_ and 41.881 pg/mL for p-Tau_181_

*APOE* apolipoprotein, *K-MMSE* Korean version of the Mini-Mental State Examination, *GDS* Geriatric Depression Scale, *CDR* Clinical dementia rating, *Aβ*_*1–42*_ amyloid β_(1–42)_, *p-Tau*_*181*_ phosphorylated tau, *t-Tau* total tau protein, *MTL* medial temporal lobe, *ROI* region of interest, *L* left, *R* right, *HC* hippocampus volume, *EC* entorhinal cortex thickness

^a^Missing data for one subject

^b^Missing data for 11 subjects

^*^Significantly different between the indicated group and the A-/T- group

^†^Significantly different between the A+/T- and A+/T+ groups

### Neuropsychological characteristics

Neuropsychological characteristics are shown in Table 2. RCFT imm, RCFT delayed, RCFT rec, and SVLT rec scores were significantly lower in the A+T+ group than in the A-T- and A+T- groups. SVLT imm and SVLT delayed scores were lower in the A+T+ group than in the A-T- group. RCFT copy score was lower in the A+T+ group than in the A+T- group (Table 2). After Bonferroni correction, three memory scores from the RCFT and two scores from the SVLT remained significant. However, no group differences were found in the test scores for psychomotor speed, attention, language, and executive function. A subset of participants who completed whole memory tests including LM (A-T- = 80, A+T- = 14, A+T+ = 19) also showed similar results on the RCFT and SVLT. In contrast, there were no differences in LM I, LM II, and LM rec scores among the groups (Table 2).

**Table 2:** Group difference in neuropsychological test scores

	A-/T-^**a**^	A+/T-^**b**^	A+/T+^**c**^	***p*** value	Post hoc
**Psychomotor speed**					
TMT A	37.51 ± 2.60	31.40 ± 5.00	39.50 ± 3.24	0.395	n.s
**Attention**					
DSF	5.41 ± 0.14	5.67 ± 0.27	5.76 ± 0.17	0.278	n.s
DSB	3.22 ± 0.10	3.67 ± 0.19	3.45 ± 0.12	0.083	n.s
**Language**					
BNT	43.63 ± 0.98	45.29 ± 1.91	42.62 ± 1.22	0.497	n.s
**Visuospatial function**					
RCFT copy	31.02 ± 0.62	33.14 ± 1.20	28.75 ± 0.75	0.006	b > c
**Memory**					
SVLT imm	17.20 ± 0.46	15.71 ± 0.89	15.12 ± 0.55	0.020	a > c
SVLT delayed	4.81 ± 0.22	3.29 ± 0.43	2.71 ± 0.27	1.5E− 7^†^	a > b, c
SVLT rec	19.90 ± 0.25	19.26 ± 0.48	17.87 ± 0.30	1.1E− 5^†^	a, b > c
LM I	15.04 ± 0.65	13.50 ± 1.54	12.25 ± 1.41	0.200	n.s
LM II	10.43 ± 0.68	9.38 ± 1.60	8.02 ± 1.46	0.343	n.s
LM rec	19.25 ± 0.51	17.54 ± 1.20	16.51 ± 1.09	0.070	n.s
RCFT imm	13.71 ± 0.66	12.56 ± 1.27	6.22 ± 0.79	2.8E− 10^†^	a, b > c
RCFT delayed	13.49 ± 0.65	12.69 ± 1.25	5.66 ± 0.79	2.3E− 11^†^	a, b > c
RCFT rec	18.92 ± 0.21	18.48 ± 0.40	17.19 ± 0.25	6.0E− 6^†^	a, b > c
**Executive function**					
Fluency A	13.35 ± 0.43	13.06 ± 0.82	11.70 ± 0.51	0.062	n.s
Fluency P	21.44 ± 0.85	22.68 ± 1.65	20.15 ± 1.06	0.402	n.s
Stroop	71.54 ± 2.27	63.13 ± 4.37	67.57 ± 2.79	0.201	n.s
TMT B	68.12 ± 5.16	89.58 ± 11.03	75.09 ± 7.11	0.196	n.s

Data are presented as means ± standard deviations. A and T classification system, for “A” (based on the value of CSF Aβ_1–42_), and “T” (based on the value of CSF p-Tau_181_). The following CSF thresholds were used: 385.822 pg/mL for Aβ_1–42_, and 41.881 pg/mL for p-Tau_181_

*TMT* trail making test, *DSF* digit span forward, *DSB* digit span backward, *BNT* Boston naming test (15 item), *RCFT copy* Rey complex figure test copy score, *SVLT imm* Seoul verbal learning test immediate recall score, *SVLT delayed* SVLT delayed recall score, *SVLT rec* SVLT recognition score, *LM I* Logical Memory immediate recall score, *LM II* Logical Memory, delayed recall score, *LM rec* Logical Memory recognition score, *RCFT imm* RCFT immediate recall score, *RCFT delayed* RCFT delayed recall score, *RCFT rec* RCFT, recognition score, *Fluency A* fluency score for animal, *Fluency P* fluency score for 3 Korean letters, *Stroop* Stroop score for color naming in color-word in incongruent condition

^a^A-/T-

^b^A+/T-

^c^A+/T+

^†^Significant group difference at the Bonferroni corrected level, *p* < 0.0028

### Relationship between p-Tau level and cognitive function

Partial correlation of p-Tau level with neuropsychological scores controlling for demographic, K-MMSE, APOE ε4 positivity, and Aβ_1–42_ levels showed a significant association between visuospatial memory scores and p-Tau level (e.g., RCFT imm, − 0.285, *p* = 0.007; RCFT delayed, − 0.254, *p* = 0.017; and RCFT rec, − 0.209, *p* = 0.051). For verbal memory scores, however, weaker (SVLT rec) or no associations (SVLT imm, SVLT delayed) were observed (Table 3). None of the other neuropsychological test scores showed a significant correlation with p-Tau level. Hierarchical multiple linear regression analysis revealed that the RCFT delayed score was the only significant predictor for p-Tau level after controlling for demographic, K-MMSE, APOE ε4 positivity, and Aβ_1–42_ levels (Table 4). In addition, all three biomarkers were included in a regression model to test whether Aβ_1–42_ and t-Tau levels also contribute to such memory measures. Both p-Tau (*β* = − 0.329, *p* < 0.0001 for RCFT delayed; *β* = − 0.191, *p* = 0.004 for SVLT delayed) and Aβ_1–42_ (*β* = 0.163, *p* = 0.027 for RCFT delayed; *β* = 0.190, *p* = 0.008 for RCFT delayed) were significant, whereas t-Tau was insignificant (Supplementary table S3 and S4).

**Table 3:** Partial correlations of p-Tau with neuropsychological scores

Neuropsychological tests	p-Tau	
	***r***	***p*** value
**Psychomotor speed**		
TMT A	− 0.019	0.862
**Attention**		
DSF	0.052	0.630
DSB	− 0.205	0.056
**Language**		
BNT	− 0.001	0.991
**Visuospatial function**		
RCFT copy	− 0.185	0.084
**Memory**		
SVLT imm	0.014	0.898
SVLT delayed	− 0.089	0.407
SVLT rec	− 0.226	0.034
LM I	− 0.134	0.213
LM II	− 0.063	0.562
LM rec	− 0.013	0.906
RCFT imm	− 0.285	0.007
RCFT delayed	− 0.254	0.017
RCFT rec	− 0.209	0.051
**Executive function**		
Fluency A	− 0.070	0.519
Fluency P	0.067	0.537
Stroop	0.115	0.286
TMT B	− 0.057	0.601

Partial correlation was performed controlling for age, sex, education, Korean version of the Mini-Mental State Examination, apolipoprotein genotype, and amyloid-β_(1–42)_

*p-Tau* phosphorylated tau, *TMT* trail making test, *DSF* digit span forward, *DSB* digit span backward, *BNT* Boston naming test (15 item), *RCFT copy* Rey complex figure test copy score, *SVLT imm* Seoul verbal learning test immediate recall score, *SVLT delayed* SVLT delayed recall score, *SVLT rec* SVLT recognition score, *LM I* Logical Memory immediate recall score, *LM II* Logical Memory, delayed recall score, *LM rec* Logical Memory recognition score, *RCFT imm* RCFT immediate recall score, *RCFT delayed* RCFT delayed recall score, *RCFT rec* RCFT, recognition score, *Fluency A* fluency score for animal, *Fluency P* fluency score for 3 Korean letters, *Stroop* Stroop score for color naming in color-word in incongruent condition

**Table 4:** Hierarchical multiple linear regression analysis for neuropsychological tests associated with p-Tau

Independent variables	Step 1^**a**^			Step 2^**b**^		
	***B***	***SE B***	***β***	***B***	***SE B***	***β***
Constant	90.685^**^	12.332		82.134**	11.764	
Sex	2.953	2.435	0.087	4.139	2.309	0.122
Age	− 0.244	0.149	− 0.106	− 0.274	0.140	− 0.119
Education	− 0.220	0.252	− 0.065	− 0.146	0.238	− 0.043
APOE ε4	1.119	2.456	0.033	0.949	2.316	0.028
K-MMSE	− 0.680^*^	0.290	− 0.180	− 0.146	0.295	− 0.039
Aβ_1–42_	− 0.042^**^	0.007	− 0.423	− 0.031**	0.007	− 0.314
**RCFT delayed**				− 0.754**	**0.158**	**− 0.363**

*K-MMSE* Korean version of the Mini-Mental State Examination, *Aβ*_*1–42*_ amyloid β _(1–42),_ *p-Tau* phosphorylated tau, *RCFT delayed* Rey complex figure test delayed recall score

^a^*R*^*2*^ = 0.305, *F* = 12.664^**^

^b^*R*^*2*^ = 0.386, Δ*R*^*2*^ = 0.081, *F* = 15.441, Δ *F* = 22.642**

^*^p < 0.05

^**^p < 0.001

### Mediating role of MTL atrophy

**Fig. 2:**
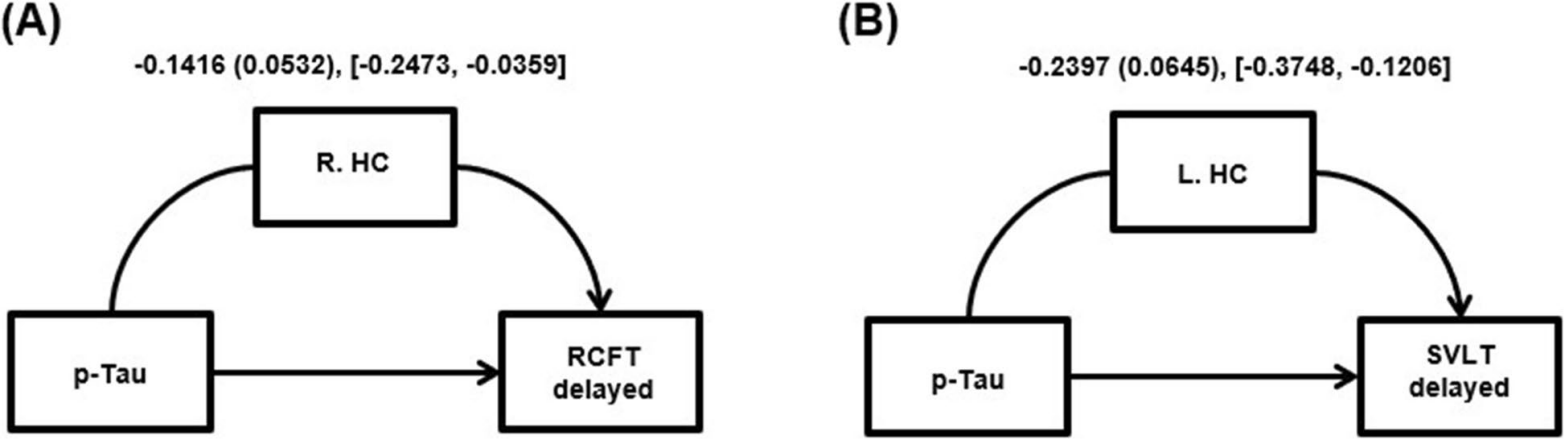
Hippocampus atrophy mediates the relationship between p-Tau level and memory scores. **A** The mediation of the relationship between p-Tau level and RCFT delayed score by right hippocampus volume. **B** The mediation of the relationship between p-Tau level and SVLT delayed score by left hippocampus volume. Values: Effect (BootSE), [BootLLCI, BootULCI]. p-Tau, phosphorylated tau; RCFT, delayed Rey complex figure test delayed recall score; SVLT, delayed Seoul verbal learning test delayed recall score; R, HC right hippocampus volume; L, HC left hippocampus volume

To test whether MTL atrophy plays a mediating role in the association between p-Tau and memory function, multiple mediation analyses were performed on delayed recall scores. The direct effect of p-Tau level on the RCFT delayed score was significant (effect = − 0.1371, *p* < 0.00001), and the right HC-mediated (indirect) effect of p-Tau level was significant for the RCFT delayed score (Fig. 2A). However, left HC did not mediate the association between p-Tau level and the RCFT delayed score. The direct effect of p-Tau level on the SVLT delayed score was also significant (effect = − 0.0293, *p* = 0.0112) and the left HC-mediated (indirect) effect of p-Tau level was significant for the SVLT delayed score (Fig. 2B). Right HC did not mediate the association between the two. Bilateral EC showed no mediating effect on the association between p-Tau level and either of the two memory scores. Additionally, MRI ROIs outside MTL (lingual gyrus, thalamus, and orbitofrontal areas) showed no significant mediating role between p-Tau level and memory scores (Supplementary Table S5).

### Development of prediction models for abnormal tau accumulation

For simplicity of the model, predictors were entered after demographic and ICV adjustments. In the first step, we tested one candidate model with RCFT delayed, which was based on the results of the hierarchical multiple linear regression analysis. RCFT delayed alone significantly predicted abnormal tau accumulation with 78.2% classification accuracy (Table 5, model A). In the second step, we tested several candidate models. Bilateral HC and bilateral EC were entered into each model (models B and C, respectively). Model A showed significantly smaller -2LL than the models with HC and EC, demonstrating better predictability (Table 5, models B and C). In addition, logistic regression with the forward conditional method was performed in order to develop optimal two candidate models among RCFT delayed, bilateral HC, and bilateral EC. Consequently, RCFT delayed (*B* = − 1.314, *p* < 0.0001, odds ratio = 0.269, 95% CI = 0.161–0.448) and left EC (*B* = − 3.190, *p* < 0.0001, odds ratio = 0.041, 95% CI = 0.010–0.174) were selected, and they significantly predicted abnormal tau accumulation with 85.6% classification accuracy (Table 5, model D). The combination of RCFT delayed and left EC showed higher classification accuracy and significantly lower -2LL than model A. In the third step, left HC was added to model D, but there was no significant -2LL difference between models D and E. Therefore, models A and D were finally selected. The prediction equation is as follows:

$$\begin{aligned}\mathrm{Model}\ \mathrm{A}:Y&=-1.952+\left(-1.536\times \mathrm{RCFT}\ \mathrm{delayed}\right)\\ \mathrm{Model}\ \mathrm{D}:Y&=12.406+\left(-1.314\times \mathrm{RCFT}\ \mathrm{delayed}\right)+\left(-3.190\times \mathrm{left}\ \mathrm{EC}\right)\end{aligned}$$

**Table 5:** Logistic regression analysis to select appropriate models for abnormal tau accumulation prediction

Models^**a**^	Classification accuracy (%)	-2 LL	χ^**2**^	df	***p*** value	Significant test for -2LL difference
**One candidate model**						
Model A: RCFT delayed	78.2	150.171	76.472	1	< 0.001	
**Two-candidate model**						
Model B: R.HC + L. HC	79.3	161.510	65.133	2	< 0.001	Model A vs. B: *p* = 0.0009
Model C: R.EC + L. EC	75.9	162.299	64.344	2	< 0.001	Model A vs. C: *p* = 0.0005
Model D: RCFT delayed + L.EC	85.6	123.479	103.163	2	< 0.001	Model A vs. D: *p*<0.001
**Three-candidate model**						
Model E: RCFT delayed + L.HC + L. EC	88.5	120.828	105.814	3	< 0.001	Model D vs. E: *p* = 0.1069

*RCFT delayed* Rey complex figure test delayed recall score, *R. HC* right hippocampus volume, *L HC* left hippocampus volume, *L. EC* left entorhinal cortex thickness

^a^Variables were adjusted for age, sex, and education, and ICV adjustment was added for HC volume and EC thickness

As shown in Table 6, the area under the curve (AUC) of the ROC curve using prediction models A and D were 0.872 and 0.921, respectively, indicating good discrimination between T- and T+. These equations were applied to the validation dataset. The AUC of the ROC curve in the validation dataset for models A and D were 0.879 and 0.891, respectively (Table 6). Additional ROC curve analysis on clinical status revealed that our models also discriminate between CN and AD (AUC = 0.956 for model D) and between MCI and AD (AUC = 0.825 for model D) (Supplementary Table S6).

**Table 6:** AUCs of models A and D in study dataset and validation dataset

Predictor	Study dataset		Validation dataset	
	AUC (SE)	95% CI	AUC (SE)	95% CI
**Model A: RCFT delayed**	0.872 (0.028)^*^	0.818–0.925	0.879 (0.046) ^*^	0.788–0.969
*Y* =− 1.952 + (− 1.536 × RCFT delayed)				
**Model D: RCFT delayed +L. EC**	0.921 (0.024)^*^	0.874–0.969	0.891 (0.052) ^*^	0.789–0.993
*Y* = 12.406 + (− 1.314 × RCFT delayed) + (− 3.190 × L. EC)				

*RCFT delayed* Rey complex figure test delayed recall score, *L. EC* left entorhinal cortex thickness

^*^p < 0.001

## Discussion

This study investigated whether tau accumulation is associated with particular cognitive impairment across the AD continuum and whether MTL atrophy mediates this association. We also developed and validated optimal prediction models for abnormal tau accumulation. We found an association between decreased visuospatial delayed memory performance and increased p-Tau levels. Temporal lobe neurodegeneration mediates the association between the two. Prediction models with visuospatial delayed memory and EC thickness for abnormal tau accumulation were validated. Preliminary findings from the current study might provide important insights into the association between tau pathology and cognitive symptoms in the AD continuum.

Cognitive impairment is a core clinical feature of the AD continuum. Given that tau protein accumulation, not Aβ burden, is highly associated with AD-related clinical symptoms [13–15], understanding the independent influence of tau accumulation on cognitive function is important for early detection and developing interventions in AD. This study found tau-associated decreased episodic memory performance that was independent of other relevant variables such as Aβ pathology level, APOE ε4 positivity, and global cognitive function. Additional analyses to test relative contributions of Aβ_1–42_, p-Tau and t-Tau on the memory scores revealed that p-Tau was the most significantly associated with memory measures (Supplementary table S3 and S4). These findings are in line with the notion that pathologic tau is a primary factor in AD-related memory change [13]. Most notably, such associations were observed mainly in the visuospatial modality of episodic memory. Our result is largely consistent with those of previous studies showing associations between tau pathology and episodic memory [8, 11, 16, 17]. However, few studies have included visuospatial episodic memory measures or controlled for other possible confounding variables, such as Aβ pathology or APOE genotype. The current results extend those of previous studies in that we included comprehensive neuropsychological assessments covering major cognitive domains; therefore, we preliminarily found tau-associated visuospatial memory impairment. In comparison with the verbal memory test score (SVLT delayed score), the visuospatial memory test score (RCFT delayed score) has a wider range of scores (SVLT delayed 0–12 vs. RCFT delayed 0–36) and higher test difficulty (recall of a simple 12-word list vs. recall of both correct positions and visually complex figures of 18 items). Therefore, we performed additional analyses on the subgroup who completed more difficult verbal memory tests, including LM testing, to rule out the possibility of the contribution of features of the neuropsychological test tool itself to the current results. Because the LM test has much higher difficulty (two stories with 25 units each) and a wider score range (0–50) than the SVLT delayed test, we assumed that the LM II test (the delayed recall part) might comparable the RCFT delayed test. We found that the LM II score had no significant association with the p-Tau level, but the RCFT delayed score did from the subgroup analysis. However, the results should be interpreted with caution, because LM II test were only available for a subset of participants. Only two previous studies included visuospatial modality of memory test [11, 17], and their results were in line with ours, suggesting a stronger association with visuospatial episodic memory than with verbal modality. Animal studies have also found that tau hyperphosphorylated mice show impaired spatial learning ability [50]. Our preliminary findings suggest that tau accumulation underlies episodic memory impairments, especially visuospatial modality.

On the other hand, we did not find any link between p-Tau and other cognitive domains, such as executive function, attention, and language. This may be partly due to the considerably milder clinical severity in our study sample. Of all the participants in our study, 59.5% had a CDR of 0.5, and only 12% had a CDR of 1. Tau associations with the non-memory domain may appear at a later stage of AD. Inclusion of more progressed cases of AD would provide a clearer picture of this issue.

Brain atrophy could be at least partially attributable to downstream of tau pathology [22, 25]. We focused on MTL areas because these regions are vulnerable to the initial tau pathology process [51]. It is highly unlikely that MTL atrophy in our dataset was caused by other etiology other than AD, because we exclude individuals with potential non-AD pathologic changes. Mediation analysis revealed that HC volume, not EC thickness, mediated the association between p-Tau levels and memory performance. Notably, the mediation effect of HC volume was observed in a modality-specific manner. Right HC-mediated indirect effect was found in visuospatial delayed memory performance, while left HC volume-mediated indirect effect was found in verbal delayed memory performance. Together, previous reports [26, 52, 53] and our findings suggest that although various processes could influence brain atrophy, tau accumulation could partly lead to neuronal loss in HC, and consequently cause episodic memory problems.

CSF tau accumulation precede cognitive decline [2, 26]. Sometimes, cognitive profiles were not sensitive enough to define AD [1] at earlier stage of disease. Prediction of abnormal tau accumulation may provide prognosis even before cognitive deficits were clinically apparent or when cognitive changes were so subtle that it cannot captured by cognitive profile. Although tau accumulation is clinically valuable information, measuring it is not always feasible, especially in community-based settings. However, no studies have demonstrated a prediction model for abnormal tau accumulation. Therefore, we proposed prediction models to be used as a screening tool in a practical way, and validated them in an independent sample. Our results from a series of logistic regression analyses showed that the RCFT delayed score alone could predict abnormal tau accumulation efficiently. Moreover, the “RCFT delayed score only” model showed better predictability than models with MTL ROIs. This result provides preliminary evidence that abnormal tau accumulation might be more sensitively detected by the lower visuospatial memory performance than by MTL atrophy. This may be plausible, especially when people are in the early stages of clinical AD, as in our study sample. RCFT can be administered simply with pencil and paper and has reported to be clinically useful in geriatric population [27, 54, 55]. However, the test has a rather complicated scoring system and strongly influenced by age, gender, and intelligence or education [54, 56]. Therefore, the test should be carefully scored and interpreted based on normative information.

Furthermore, the combination of RCFT delayed score and left EC thickness revealed an additional effect, resulting in higher classification accuracy than the RCFT delayed score alone. In line with our result, left side of EC was reported to be thinner in MCI group than CN group [57], or thinner in progressive (to AD) MCI group than stable MCI group [58]. The ROC curve analysis using these two prediction equations demonstrated significant discrimination ability between T positive and T negative individuals in the independent validation sample as well as in the current study sample. Additionally, the suggested models also seem to be useful to discriminate AD from CN or MCI, but not MCI from CN.

Tau accumulation can be evaluated by either CSF or PET methods. In the current study we adopted CSF method, because CSF analysis can straightforwardly assess in vivo AD pathophysiology [59]. Specifically, elevated p-Tau level is a direct biomarker of fibrillar tau. It best reflects the pathologic state at the time of the test that is associated with AD core pathology. CSF p-Tau denotes an ongoing active pathologic state and detects earlier pathological changes than PET [2, 60, 61]. However, it is worth mentioning that tau PET can also play a role as a state marker for cognitive decline in AD [8, 62]. Tau PET measures the magnitude of pathological tau accumulation over time [1].

### Limitations

Despite its significant implications, the current study has limitations and future directions to be discussed. First, this study used a cross-sectional design; therefore, the results should be interpreted with caution. Further studies with longitudinal follow-up could provide a better understanding of the actual cognitive consequences of abnormal tau accumulation. Second, sample size in the A+/T- subgroups (including validation sample) and in CN individuals with AD pathology-positive, i.e., preclinical stage, are very small. Larger sample size in such subgroups could shed more light on the association between abnormal tau accumulation and cognitive function in the initial process of the disease and provide more clinical implication. Lastly, our data provide preliminary evidence that visuospatial episodic memory tests could sensitively reflect abnormal tau accumulation. Given that verbal episodic memory tests are mainly used and the visuospatial tests are far less used in the field, it is certainly one area for additional research that could enhance the understanding of the nature of visuospatial episodic memory

## Conclusions

In conclusion, it is crucial to identify sensitive cognitive measures that capture subtle cognitive impairment associated with underlying pathological changes. Preliminary findings from the current study might suggest that abnormal tau accumulation underlies episodic memory impairment, particularly visuospatial modality, in the AD continuum. Suggested models are potentially useful in predicting tau pathology and might be utilized practically in the field.

## Supplementary Information

**Additional file 1: Supplemental Table S1.** Demographic and clinical characteristics of clinical diagnosis subgroups. Data are presented as means ± standard deviations, unless specified otherwise. A and T classification system, for “A” (based on the value of CSF Aβ_1–42_), and “T” (based on the value of CSF p-Tau_181_). The following CSF thresholds were used: 385.822 pg/mL for Aβ_1–42_, and 41.881 pg/mL for p-Tau_181_. **Supplemental Table S2.** Clinical characteristics in validation data set. Data are presented as means ± standard deviation, unless specified otherwise. The following CSF thresholds were used: 385.822 pg/mL for Aβ_1–42_, and 41.881 pg/mL for p-Tau_181_. **Supplemental Table S3.** Partial correlations of Aβ_1–42_. and t-Tau with neuropsychological scores. Partial correlation was performed controlling for age, sex, education, Korean version of the Mini-Mental State Examination, apolipoprotein genotype, and CSF biomarkers. **Supplemental Table S4.** Multiple linear regression analysis with stepwise selection. Multiple linear regression was performed controlling for age, sex, education, Korean version of the Mini-Mental State Examination, and apolipoprotein genotype. **Supplemental Table S5.** Mediation analysis of MRI control regions of interests. Data are presented as effect (BootSE), [BootLLCI, BootULCI]. **Supplemental Table S6.** AUCs of models A and D in CN, MCI, and AD groups.

## Data Availability

The datasets analyzed for the current study are not publicly available but are available from the corresponding author on reasonable request.

## Abbreviations

AD: Alzheimer’s disease
APOE: The apolipoprotein
AUC: The area under the curve
Aβ: Amyloid-beta
CDR: A clinical dementia rating
CN: Cognitively normal
CSF: Cerebrospinal fluid
EC: Entorhinal cortex
GARD: The Gwanju Alzheimer’s Disease and Related Dementia
HC: Hippocampus
ICV: Intracranial volume
KBASE-V: The Korean Brain Aging Study for Early Diagnosis and Prediction of AD
K-MMSE: Korean version of the Mini-Mental State Examination
LM: The Logical Memory
LM rec: Delayed recognition
LMI: Immediate recall
LMII: Delayed recall
MCI: Mild cognitive impairment
MRI: Magnetic resonance imaging
MTL: The medial temporal lobe
NFT: Neurofibrillary tangles
PET: Positron emission tomography
p-Tau: Phosphorylated tau
RCFT copy: The Rey complex figure test
RCFT delayed: The RCFT immediate recall 20-min delayed recall
RCFT imm: The RCFT immediate recall
RCFT rec: The RCFT immediate recall yes-no recognition
ROC: Receiver operating characteristic
SVLT: The Seoul Verbal Learning Test
SVLT delayed: The Seoul Verbal Learning Test 20-min delayed recall
SVLT imm: The Seoul Verbal Learning Test immediate recall
SVLT rec: The Seoul Verbal Learning Test yes-no recognition
TMT: Trail making tests
t-Tau: Total tau protein
