# Supplementary material for: Visuospatial memory impairment as a potential neurocognitive marker to predict tau pathology in Alzheimer’s continuum
Source: Alzheimers Res Ther. 2021 Oct 9;13:167. doi: 10.1186/s13195-021-00909-1 (PMC8502282; doi:10.1186/s13195-021-00909-1)
Supplement: Supplementary file 1 — Additional file 1: Supplemental Table S1. Demographic and clinical characteristics of clinical diagnosis subgroups. Data are presented as means ± standard deviations, unless specified otherwise. A and T classification system, for “A” (based on the value of CSF Aβ1–42), and “T” (based on the value of CSF p-Tau181). The following CSF thresholds were used: 385.822 pg/mL for Aβ1–42, and 41.881 pg/mL for p-Tau181. Supplemental Table S2. Clinical characteristics in validation data set. Data are presented as means ± standard deviation, unless specified otherwise. The following CSF thresholds were used: 385.822 pg/mL for Aβ1–42, and 41.881 pg/mL for p-Tau181. Supplemental Table S3. Partial correlations of Aβ1–42. and t-Tau with neuropsychological scores. Partial correlation was performed controlling for age, sex, education, Korean version of the Mini-Mental State Examination, apolipoprotein genotype, and CSF biomarkers. Supplemental Table S4. Multiple linear regression analysis with stepwise selection. Multiple linear regression was performed controlling for age, sex, education, Korean version of the Mini-Mental State Examination, and apolipoprotein genotype. Supplemental Table S5. Mediation analysis of MRI control regions of interests. Data are presented as effect (BootSE), [BootLLCI, BootULCI]. Supplemental Table S6. AUCs of models A and D in CN, MCI, and AD groups. [file 13195_2021_909_MOESM1_ESM.docx]

**Supplementary Table S1. Demographic and clinical characteristics of clinical diagnosis subgroups**

|  | **CN** | **MCI** | **AD** | ***p* value** |
| --- | --- | --- | --- | --- |
|  | **(*n* = 49)** | **(*n* = 96)** | **(*n* = 40)** |  |
| Age, y | 71.0 ± 5.1 | 71.2 ± 7.2 | 66.6 ± 9.3^*^*^†^* | .002 |
| Education, y | 10.3 ± 5.4 | 10.8 ± 4.8 | 7.0 ± 3.9^*^*^†^* | < .001 |
| Female (n, %) | 23 (46.9) | 45 (46.9) | 27 (67.5) | .070 |
| APOE ε4 carrier (n, %) | 11 (22.4) | 37 (38.5) | 27 (69.2) | < .001 |
| GDS | 8.0 ± 6.2 | 11.6 ± 6.8^*^ | 12.6 ± 6.0^*^ | .005 |
| K-MMSE | 27.4 ± 2.3 | 25.8 ± 3.0^*^ | 19.5 ± 5.4^*^*^†^* | < .001 |
| CDR SOB | 0.1 ± 0.2 | 0.9 ± 0.7^*^ | 3.9 ± 2.0^*^*^†^* | < .001 |
| CSF biomarkers (pg/ml) |  |  |  |  |
| Aβ_1-42_ | 467.0 ± 150.3 | 385.9 ± 172.7^*^ | 238.3 ± 89.8^*^*^†^* | < .001 |
| p-Tau | 31.4 ± 8.7 | 38.4 ± 15.1^*^ | 59.4 ± 17.2^*^*^†^* | < .001 |
| t-Tau | 51.4 ± 15.8 | 66.1 ± 32.3^*^ | 108.9 ± 49.0^*^*^†^* | < .001 |
| A-T-/ A+T-/ A+T+ (*n*) | 40/ 6/ 3 | 53/ 12/ 31 | 0/ 5/ 35 | < .001 |

Data are presented as means ± standard deviations, unless specified otherwise. A and T classification system, for “A” (based on the value of CSF Aß_1-42_), and “T” (based on the value of CSF p-Tau_181_).

* Comparison between the indicated group and the CN group; *†* Comparison between the MCI and AD group.

Abbreviations: *APOE* apolipoprotein, *K-MMSE* Korean version of the Mini-Mental State Examination, *GDS* Geriatric Depression Scale, *CDR SOB* Clinical dementia rating sum of boxes, *Aß_1-42_* amyloid β_(1-42)_, *p-Tau_181_* phosphorylated tau, *t-Tau* total tau protein,

**Supplementary Table S2. Clinical characteristics in validation data set**

|  | **A-/T-** | **A+/T-** | **A+/T+** | ***p* value** |
| --- | --- | --- | --- | --- |
|  | **(*n* = 49)** | **(*n* = 5)** | **(*n* = 17)** |  |
| Age | 64.6 ± 6.6 | 74.8 ± 3.5^*^ | 72.5 ± 8.1^*^ | <0.001 |
| Education | 9.5 ± 5.1 | 8.8 ± 3.4 | 9.3 ± 3.8 | 0.944 |
| Female (n, %) | 31 (63.3) | 2 (40.0) | 7 (41.2) | 0.214 |
| APOE ε4 carrier,^a^ n (%) | 7 (14.3) | 2 (40.0) | 11 (64.7) | <0.001 |
| MMSE | 27.0 ± 2.6 | 21.0 ± 5.8^*^ | 17.9 ± 4.9^*^ | <0.001 |
| GDS | 8.2 ± 6.9 | 16.2 ± 8.0 | 9.8 ± 7.6 | 0.061 |
| CDR 0, n (%) | 49 (100.0) | - | - | <0.001 |
| 0.5, n (%) | - | 4 (80.0) | 10 (58.8) |  |
| 1, n (%) | - | 1 (20.0) | 7 (41.2) |  |
| CSF biomarkers (pg/ml) |  |  |  |  |
| Aß_1-42_ | 554.4 ± 52.1 | 312.1 ± 90.1^*^ | 266.4 ± 62.5^*^ | <0.001 |
| p-Tau_181_ | 15.3 ± 3.1 | 15.9 ± 3.6 | 44.0 ± 21.1^*^*^†^* | <0.001 |
| t-Tau | 47.3 ± 7.4 | 41.1 ± 4.7 | 100.0 ± 34.8^*^*^†^* | <0.001 |

Data are presented as means ± standard deviation, unless specified otherwise. A and T classification system, for “A” (based on the value of CSF Aß_1-42_), and “T” (based on the value of CSF p-Tau_181_).

Abbreviations: *APOE* apolipoprotein, *K-MMSE* Korean version of the Mini-Mental State Examination, *GDS* Geriatric Depression Scale, *CDR* Clinical dementia rating, *Aß_1-42_* amyloid β_(1-42)_, *p-Tau_181_* phosphorylated tau, *t-Tau* total tau protein,

**Supplementary Table S3.** Partial correlations of Aß_1-42._ and t-Tau with neuropsychological scores ^a^

| **Neuropsychological tests** | Aß_1-42_ | | t-Tau | |
| --- | --- | --- | --- | --- |
|  | ***r*** | ***p* value** | ***r*** | ***p* value** |
| **Psychomotor speed** |  |  |  |  |
| TMT A | 0.006 | 0.953 | 0.071 | 0.449 |
| **Attention** |  |  |  |  |
| DSF | -0.190 | 0.041 | -0.002 | 0.982 |
| DSB | -0.109 | 0.244 | -0.072 | 0.442 |
| **Language** |  |  |  |  |
| BNT | 0.210 | 0.024 | -0.208 | 0.025 |
| **Visuospatial function** |  |  |  |  |
| RCFT copy | -0.031 | 0.744 | 0.054 | 0.568 |
| **Memory** |  |  |  |  |
| SVLT imm | 0.133 | 0.154 | -0.028 | 0.763 |
| SVLT delayed | 0.153 | 0.100 | -0.150 | 0.108 |
| SVLT rec | 0.066 | 0.484 | -0.173 | 0.064 |
| RCFT imm | 0.096 | 0.308 | 0.004 | 0.962 |
| RCFT delayed | 0.063 | 0.500 | -0.026 | 0.785 |
| RCFT rec | 0.139 | 0.136 | -0.174 | 0.062 |
| **Executive function** |  |  |  |  |
| Fluency A | 0.126 | 0.179 | -0.120 | 0.200 |
| Fluency P | -0.007 | 0.943 | -0.096 | 0.306 |
| Stroop | 0.142 | 0.129 | -0.038 | 0.683 |
| TMT B | -0.145 | 0.120 | -0.174 | 0.062 |

^a^ Partial correlation was performed controlling for age, sex, education, Korean version of the Mini-Mental State Examination, apolipoprotein genotype, and CSF biomarkers

Abbreviations: *p-Tau* phosphorylated tau, *t-Tau* total tau protein, *TMT* trail making test, *DSF* digit span forward, *DSB* digit span backward, *BNT* Boston naming test (15 item), *RCFT copy* Rey complex figure test copy score, *SVLT imm* Seoul verbal learning test immediate recall score, *SVLT delayed* SVLT delayed recall score, *SVLT rec* SVLT recognition score, *RCFT imm* RCFT immediate recall score, *RCFT delayed* RCFT delayed recall score, *RCFT rec* RCFT, recognition score, *Fluency A* fluency score for animal, *Fluency P* fluency score for 3 Korean letters, *Stroop* Stroop score for color naming in color-word in incongruent condition

**Supplementary Table S4.** Multiple linear regression analysis with stepwise selection

| **Independent variables** | **RCFT delayed**^a^ | | | |  | **SVLT delayed**^b^ | | | |
| --- | --- | --- | --- | --- | --- | --- | --- | --- | --- |
|  | ***B*** | ***SE B*** | ***β*** | ***p*** |  | ***B*** | ***SE B*** | ***β*** | ***p*** |
| **p-Tau** | -0.155 | 0.032 | -0.329 | 0.000 |  | -0.032 | 0.011 | -0.191 | 0.004 |
| **Aß_1-42_** | 0.008 | 0.004 | 0.163 | 0.027 |  | 0.003 | 0.001 | 0.190 | 0.008 |

^a^ *R^2^* = 0.476, *F*= 21.574, Δ*F*=4.947^*^; *p= 0.027*

^b^ *R^2^* = 0.517, *F*= 25.263; Δ*F*=7.214^**^ *p=0.008*

Multiple linear regression was performed controlling for age, sex, education, Korean version of the Mini-Mental State Examination, and apolipoprotein genotype.

Abbreviations: *Aß_1-42_* amyloid ß _(1-42),_ *p-Tau* phosphorylated tau, *RCFT delayed* Rey complex figure test delayed recall score, SVLT delayed Seoul verbal learning test delayed recall score.

**Supplementary Table S5. Mediation analysis of MRI control regions of interests**

|  | **ROIs** | **Hemisphere** | **Effect** | **BootSE** | **BootLLCI** | **BootULCI** |
| --- | --- | --- | --- | --- | --- | --- |
| **RCFT delayed recall** | **Total** | - | -0.0177 | 0.0127 | -0.0435 | 0.0068 |
|  | **Thalamus** | L | -0.0067 | 0.0067 | -0.0227 | 0.0031 |
|  |  | R | 0.0003 | 0.0034 | -0.0064 | 0.0082 |
|  | **LING** | L | -0.0005 | 0.0085 | -0.0187 | 0.0165 |
|  |  | R | -0.0068 | 0.0080 | -0.0254 | 0.0063 |
|  | **MOFC** | L | 0.0000 | 0.0035 | -0.0063 | 0.0089 |
|  |  | R | -0.0026 | 0.0051 | -0.0146 | 0.0063 |
|  | **LOFC** | L | -0.0015 | 0.0046 | -0.0125 | 0.0070 |
|  |  | R | 0.0001 | 0.0038 | -0.0094 | 0.0069 |
| **SVLT delayed recall** | **Total** | - | -0.0021 | 0.0046 | -0.0113 | 0.0069 |
|  | **Thalamus** | L | 0.0012 | 0.0020 | -0.0019 | 0.0061 |
|  |  | R | -0.0009 | 0.0025 | -0.0069 | 0.0035 |
|  | **LING** | L | -0.0020 | 0.0030 | -0.0091 | 0.0026 |
|  |  | R | 0.0018 | 0.0028 | -0.0027 | 0.0083 |
|  | **MOFC** | L | 0.0002 | 0.0013 | -0.0021 | 0.0034 |
|  |  | R | -0.0025 | 0.0025 | -0.0083 | 0.0013 |
|  | **LOFC** | L | -0.0002 | 0.0014 | -0.0035 | 0.0025 |
|  |  | R | 0.0004 | 0.0020 | -0.0042 | 0.0044 |

Data are presented as effect size (BootSE), [BootLLCI, BootULCI].

Abbreviations: ROIs region of interests, *RCFT* delayed Rey complex figure test delayed recall score, *SVLT* delayed Seoul verbal learning test delayed recall score, LING lingual gyrus, MOFC medial orbitofrontal cortex, LOFC later orbitofrontal cortex, L left, R right.

**Supplementary Table S6. AUCs of Models A and D in CN, MCI, and AD groups**

| **Predictor** | **CN vs MCI** | |  | **CN vs AD** | |  |  | **MCI vs AD** | |
| --- | --- | --- | --- | --- | --- | --- | --- | --- | --- |
|  | **AUC (SE)** | **95% CI** |  | **AUC (SE)** | **95% CI** |  |  | **AUC (SE)** | **95% CI** |
| **Model A: RCFT delayed** | 0.731 (0.043) ^*^ | 0.647-0.814 |  | 0.944 (0.026) ^*^ | 0.894-0.995 |  |  | 0.816 (0.043) ^*^ | 0.732-0.900 |
| **Model D: RCFT delayed +L. EC** | 0.730 (0.043) ^*^ | 0.647-0.813 |  | 0.956 (0.022) ^*^ | 0.914-0.999 |  |  | 0.825 (0.041) ^*^ | 0.745-0.905 |

**p* <0.001

Abbreviations: *RCFT delayed* Rey complex figure test delayed recall score, *L. EC* left entorhinal cortex thickness, CN cognitive normal, MCI mild cognitive impairment, AD Alzheimer’s disease.
